# Molecular drivers of lobular carcinoma *in situ*

**DOI:** 10.1186/s13058-015-0580-5

**Published:** 2015-06-04

**Authors:** Greg J. Logan, David J. Dabbs, Peter C. Lucas, Rachel C. Jankowitz, Daniel D. Brown, Beth Z. Clark, Steffi Oesterreich, Priscilla F. McAuliffe

**Affiliations:** 1 Womens Cancer Research Center, University of Pittsburgh Cancer Institute, Pittsburgh, PA 15213 USA; 2 Department of Pharmacology and Chemical Biology, University of Pittsburgh, Pittsburgh, PA 15213 USA; 3 Department of Pathology, Magee-Womens Hospital, Pittsburgh, PA 15213 USA; 4 Division of Medical Oncology, Department of Medicine, University of Pittsburgh School of Medicine, Pittsburgh, PA 15213 USA; 5 Division of Surgical Oncology, Department of Surgery, University of Pittsburgh School of Medicine, Pittsburgh, PA 15213 USA

## Abstract

Lobular carcinoma *in situ* (LCIS) is considered to be a risk factor for the development of invasive breast carcinoma, but it may also be a non-obligate precursor to invasive lobular carcinoma (ILC). Many LCIS lesions do not progress to ILC, and the molecular changes that are necessary for progression from LCIS to ILC are poorly understood. Disruption in the E-cadherin complex is the hallmark of lobular lesions, but other signaling molecules, such as PIK3CA and c-src, are consistently altered in LCIS. This review focuses on the molecular drivers of lobular carcinoma, a more complete understanding of which may give perspective on which LCIS lesions progress, and which will not, thus having immense clinical implications.

## Introduction

Lobular carcinoma *in situ* (LCIS) has long been considered a risk factor for the future development, in either breast, of invasive breast carcinoma (IBC), but recent evidence suggests that LCIS may also be a non-obligate precursor to IBC, and more specifically to invasive lobular carcinoma (ILC).

LCIS is rarely detected by physical examination, nor does it have specific diagnostic mammographic findings [1]. Currently about 0.5 to 3.9 % of image-guided core needle biopsies incidentally identify LCIS and, as mammographic technology improves, the incidence of LCIS is rising [1, 2]. Patients diagnosed with LCIS have an 8- to 10-fold increased lifetime risk of developing breast cancer, compared with women without this diagnosis [3]. The likelihood of developing IBC increases by about 1 % every year after LCIS diagnosis - with a 13 % risk after 10 years and a 21 to 26 % risk after 20 years [4, 5]. In a recent subgroup analysis of participants of the Canadian National Breast Screening Study, the cumulative probability of subsequent breast cancer occurrence 5 years after diagnosis was lower for LCIS, compared with ductal carcinoma *in situ* (DCIS) (5.7 % versus 11.4 %, respectively); however, by 20 years after the diagnosis of LCIS or DCIS, rates of IBC were equivalent (21.3 % and 19 %, respectively) [5].

LCIS was originally described as ‘lobular’ because the lesions appeared most often in the terminal duct lobular units (TDLUs), whereas ductal lesions appeared most often in the mammary ducts. However, it is now understood that all pre-invasive lesions originate from the TDLUs [6–8] but the terms ‘lobular’ and ‘ductal’ have persisted.

LCIS is believed to arise from atypical lobular hyperplasia (ALH), a pre-invasive lesion with morphological features similar to LCIS, except with smaller, less distended acini. ALH and LCIS share similar chromosomal changes and molecular features [9]. Since the factors that distinguish ALH from LCIS are somewhat subjective [10], the term lobular neoplasia (LN) has been adopted by many to encompass all pre-invasive lobular disease. The most well-studied characteristic of LN is loss of E-cadherin, and this is clinically used to differentiate lobular from ductal lesions [11]. Herein, we review the studies to date that focus on the molecular mechanisms of LCIS. Gaining a better understanding of the pathways underlying LCIS and its non-obligate progression to IBC might allow the development of predictive tools that would refine the management of this challenging clinical entity.

## Lobular carcinoma *in situ* progression

Historically, the concept of LCIS as a non-obligate precursor of IBC was not well accepted. Foote and Stewart first coined the term LCIS in 1941, and subsequently published long-term follow-up of their patients with LCIS reporting a 20-year cumulative risk of subsequent carcinoma of 35 % in the ipsilateral and 25 % in the contralateral breast [12, 13]. In 1978, Haagensen and colleagues [14] reported 14-year follow-up (range 1 to 42 years) of 211 patients with LCIS. Of these, 36 (17 %) patients subsequently developed IBC: 19 in the ipsilateral and 20 in the contralateral breast. In the same year, Rosen and colleagues [15] published a 24-year follow-up of 99 patients with LCIS. Thirty-nine breast IBCs occurred in 32 out of 84 patients for whom follow-up was available. IBC occurred in the ipsilateral breast in 12 patients, the contralateral breast in 9 patients, bilaterally in 7 patients and unknown in 4 patients. These two studies posited that it was unlikely that invasive cancer in one breast progressed from a pre-invasive lesion in the opposite breast, and LCIS was, therefore, merely a risk factor for the development of breast cancer in both breasts. These results prompted many physicians to assume a conservative surgical approach to treating patients with LCIS.

**Fig. 1:**
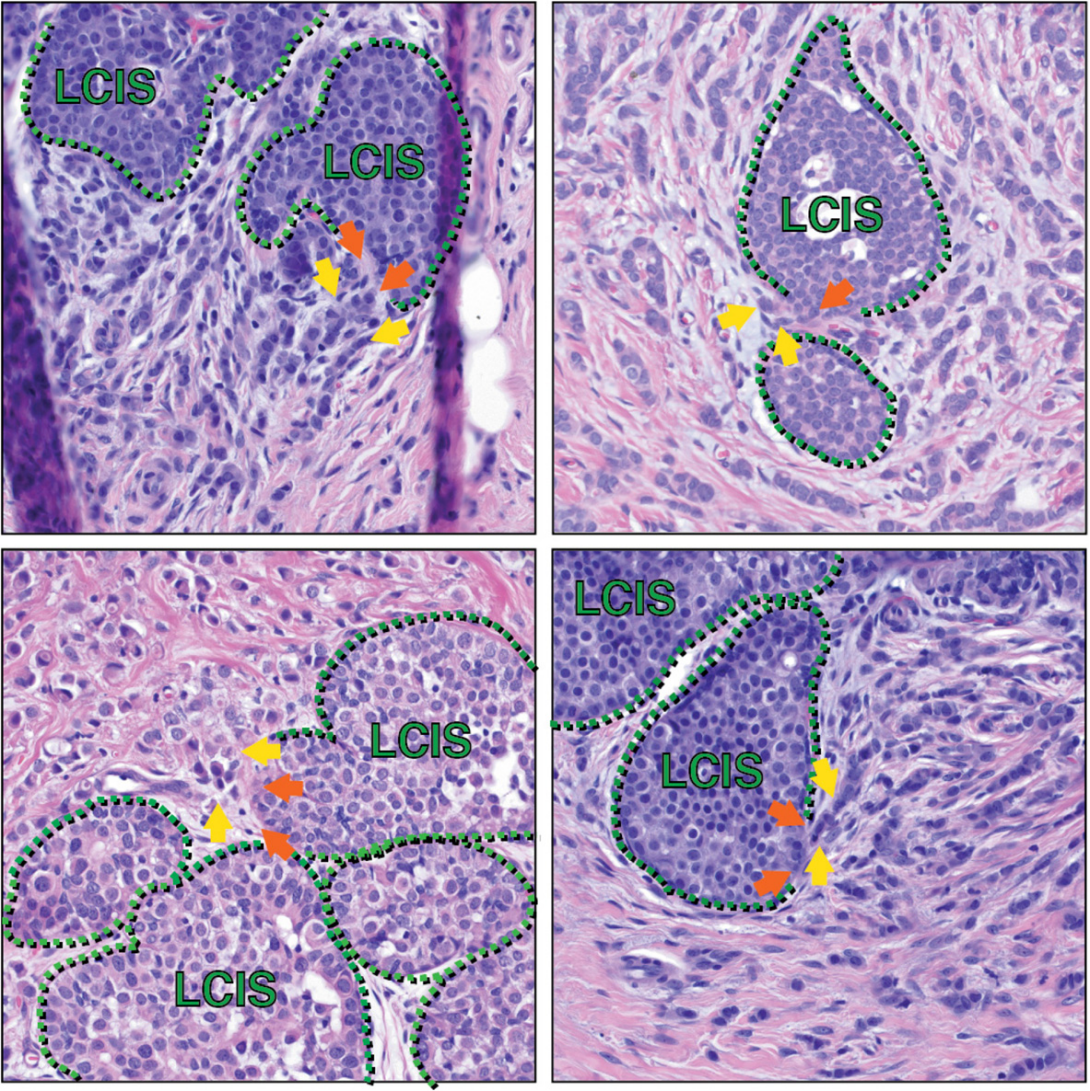
Lobular carcinoma *in situ* in association with invasive lobular carcinoma. Histological sections from multiple patients capture areas of lobular carcinoma *in situ* (LCIS) that appear to have focal myoepithelial layer disruption adjacent to invasive lobular carcinoma (ILC), suggesting possible progression of LCIS to ILC at such transitions. Green hatched lines mark the myoepithelial layer; orange arrows are possible foci of myoepithelial disruption; yellow arrows highlight invasive cells. In addition to the cells marked by yellow arrows, additional ILC cells are present in each image throughout the stroma, surrounding the areas of LCIS

In more contemporary series, however, several studies have shown a stronger propensity for development of ipsilateral IBC after diagnosis of LCIS [13, 16]. These studies, combined with genomic clonality studies comparing LCIS and IBC, support a non-obligate precursor role of LCIS, in addition to being a risk factor for IBC. Briefly, in 2003, a retrospective study by Page and colleagues [17] of 252 women, treated between 1950 and 1985, showed that IBC was 3.1 times more likely to develop in the ipsilateral breast than in the contralateral breast, after previous diagnosis of ALH. After a previous diagnosis of LCIS, IBC was two to five times more likely to develop in the ipsilateral breast [18, 19]. A study using array comparative genomic hybridization (aCGH) showed that LCIS is clonally related to synchronous IBC [20, 21]. Andrade and colleagues [22] also reached this conclusion comparing single nucleotide polymorphism (SNP) DNA microarrays of matched LCIS and synchronous lesions. Interestingly, Aulmann and colleagues [23], using mitochondrial DNA sequencing, identified some examples of clonality between LCIS and metacronous IBC (that is, an invasive breast cancer that develops in the same breast at a later time), although most cases of later breast cancer were clonally unrelated to the LCIS. Furthermore, in patients diagnosed with ILC, LCIS is often found to be closely associated. For example, in a recent study of 81 patients with ILC, 37 (46 %) had LCIS that was in close proximity to the invasive component [24]. We ourselves have frequently observed cases in which, on careful histological sectioning, there appears to be myoepithelial layer disruption at discrete foci of LCIS, accompanied by adjacent ILC, raising the possibility that these sections are capturing the transition from *in situ* to invasive disease (Fig. 1). Together, these studies support the non-obligate precursor role of LCIS.

Currently, no diagnostic tools exist which can reliably predict if a woman will subsequently develop IBC after diagnosis of LCIS. Most women with LCIS are treated conservatively, with close observation [1]. Based on the promising results of the National Surgical Adjuvant Breast and Bowel Project (NSABP) BCPT P-1 (Breast Cancer Prevention Trial) and NSABP STAR P-2 (Study of Tamoxifen and Raloxifene) trial, the most recent American Society of Clinical Oncology guidelines recommend that risk-reducing pharmacologic agents, such as tamoxifen and raloxifene, be discussed with women diagnosed with LCIS [25–27]. Additional risk factors, such as strong family history and very young age, may prompt prophylactic bilateral mastectomy, but this is pursued in only a minority of women with this diagnosis [28].

## Classification of lobular carcinoma *in situ*

Currently, histological features guide classification of LCIS lesions. The three main histological sub-classifications of LCIS are classical (CLCIS), florid (FLCIS) and pleomorphic (PLCIS), and these entities can be found to coexist.

Histologically, CLCIS is characterized by a monomorphic population of small round cells with a ring of clear cytoplasm [29]. Cells within the lesion are loosely adherent, filling the lumen of the acini and distending the TDLU, yet they maintain the architecture of the lobules with an intact basement membrane and myoepithelial cell layer [30]. Mitotic figures and necrosis, as well as calcifications, are not common in CLCIS. Pagetoid spread, in which neoplastic cells extend along the mammary ducts, is frequently observed. There are two categories of CLCIS, type A and type B [31]. Type A CLCIS is generally low grade, with small nuclei and inconspicuous nucleoli. Type B CLCIS is composed of cells with larger nuclei and small nucleoli. CLCIS tends to be positive for estrogen receptor (ER) and progesterone receptor (PR), and negative for HER2.

FLCIS is a comparatively more rare lesion, histologically characterized by massive expansion of the involved TDLUs, often associated with necrosis and calcifications. Morphologically, it resembles solid-type DCIS. The lesion is frequently associated with ILC, supporting FLCIS as a precursor of ILC [32]. FLCIS shows more genetic instability than CLCIS, including a higher fraction of genomic alterations and breakpoints [33].

PLCIS is a subtype of LCIS that is commonly associated with pleomorphic ILC and that tends to be higher grade [29, 34–36]. In contrast to CLCIS and FLCIS, the nuclei and nucleoli in PLCIS are larger, and cells have more abundant cytoplasm. Calcifications and comedo-type necrosis are more common in PLCIS than in CLCIS. PLCIS can be divided into apocrine or non-apocrine PLCIS, based on the presence or absence, respectively, of eosinophilic granules in the cytoplasm, intracytoplasmic vacuoles and vesicular chromatin [31]. Apocrine differentiation can also be marked by immunoreactivity to GCDFP-15, a protein originally isolated from breast cystic fluid and shown to be highly expressed in cancers associated with salivary glands, sweat glands, and prostate [35, 37]. The apocrine variant of PLCIS is shown to have more genetic instability, and it is the most likely to have amplified HER2 [31, 34]. Interestingly, aCGH-based data suggest that FLCIS has more genomic alterations than PLCIS as a group, but less genetic complexity than apocrine PLCIS [33].

Beyond the most common type A CLCIS, classification of LCIS is difficult and can be somewhat subjective, especially in the setting of higher nuclear grade, presence of calcifications, necrosis and/or unusal ER or HER2 status. There is also controversy regarding treatment of FLCIS and PLCIS. PLCIS is usually treated more aggressively, with surgery including re-excision to negative margins and often with radiation therapy, as well as with endocrine therapy. However, there are limited data to support clinical decision making for these entities.

An alternative classification system for LCIS was developed by Bratthauer and Tavassoli [38]. This classification uses the term lobular intraepithelial neoplasia (LIN) or LN in lieu of ALH and LCIS. Some opine that LIN grading is better because it removes the term ‘carcinoma’, which can be a confusing term in the context of *in situ* disease [39]. LIN is divided into three grades. LIN1 corresponds to ALH lesions where the lumen is filled, but the acini are not distended. LIN2 lesions have acini that are distended but not fused, corresponding to CLCIS lesions. LIN3 describes more advanced LCIS with marked distention of the lobules, including FLCIS and PLCIS.

Sub-classifying LCIS, whether by histology or by LIN grade, has been useful for identifying lesions more likely to progress so that they may be treated more aggressively [40]. Clearly, improving our understanding of molecular drivers of LCIS progression will be an important way to improve our ability to differentiate aggressive from benign premalignant lesions and further personalize treatment recommendations for patients.

## Molecular characteristics of lobular carcinoma *in situ*

**Fig. 2:**
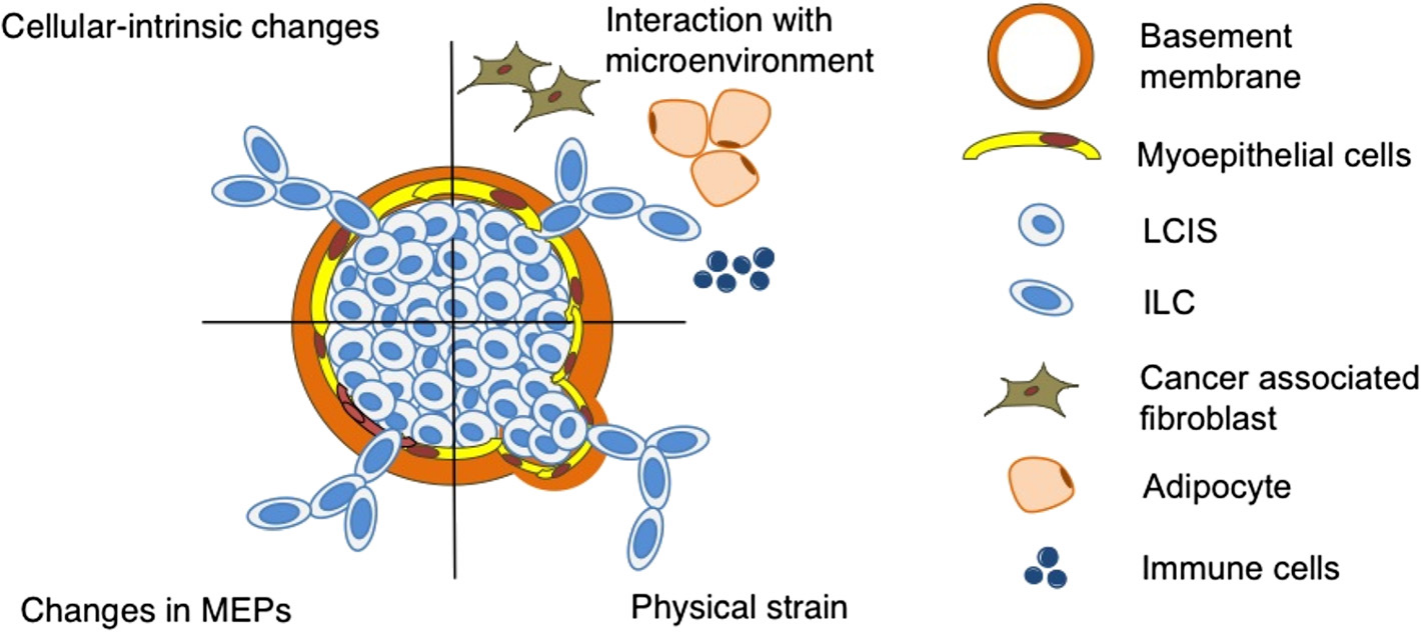
Proposed mechanisms of lobular carcinoma *in situ* progression to invasive breast cancer. Progression of lobular carcinoma *in situ* (LCIS) to invasive breast cancer may be influenced by multiple factors, including cell-intrinsic changes, such as mutations, extrinsic factors from interaction with the microenvironment, changes within the myoepithelial cells (MEPs), and physical strain on the basement membrane components, exerted by LCIS within the lobule, causing cells to rupture the lobule. ILC, invasive lobular carcinoma

In contrast to the role of LCIS as a risk factor for the development of breast cancer, a role for direct progression of LCIS into invasive cancer is less well accepted, and the molecular basis is currently poorly understood. LCIS must traverse myoepithelial cells and the basement membrane in order to invade locally. Schematically shown in Fig. 2 are four proposed mechanisms that could influence this process, and that are likely not mutually exclusive. Cells may acquire genetic (and/or epigenetic) changes in critical pathways that allow migration into the stroma. Alternatively, or in concert, cells may receive signals that cause progression from the stroma, including fibroblasts, adipocytes, and immune cells. Another possibility is that tumor suppressive myoepithelial cells may become compromised, allowing LCIS cells to break through and gain access to the stroma. Finally, enlarging LCIS cellular density may cause physical strain on the myoepithelial cells and basement membrane such that the cells may physically rupture a lobule. It is most likely that the progression of LCIS to IBC occurs through a combination of these events.

To better identify LCIS lesions that may progress versus those that remain dormant requires a thorough knowledge of the mechanisms that drive progression to invasive disease. Current research on LCIS has focused on four main areas: (1) prognostic markers, (2) genomic changes, (3) factors related to epithelial to mesenchymal transition (EMT), and (4) signaling pathways. We summarize and discuss those areas of research below.

### Prognostic markers

The expression of nuclear receptors - especially ER - can be used to predict clinical outcome of tumors [41, 42]; 80 to 100 % of LCIS cases express ERα, most of which show moderate to strong immunoreactivity by immunohistochemistry [43–46] (Table 1). Similarly, ILC is also a highly ER-positive disease, with greater than 90 % ER positivity [47, 48]. Some aggressive variants of LCIS are more likely to be ER-negative. For example, 80 % of apocrine PLCIS lesions are ER-negative [49]. This suggests that ER-negativity in LCIS may be a potential marker of progression of more aggressive lesions. However, because most CLCIS and many PLCIS lesions are ER-positive, additional prognostic markers are clearly needed to better differentiate ER-positive lesions that will progress versus those that will not.

**Table 1:** Common prognostic markers in classical lobular carcinoma *in situ*

Reference	n	ERα (%)	PR (%)	c-erbB-2 (%)
Rudas *et al*. 1997 [46]	23	80	90	4
Fisher *et al*. 1998 [26]	15	100	100	0
Querzoli *et al*. 1998 [45]	19	100	47	11
Mohsin *et al*. 2005 [44]	57	98	84	4
Chen *et al*. 2009 [49]	21	100	100	0
Green *et al*. 2009 [43]	47	100	100	
Vincent-Salomon *et al*. 2012 [59]	58	91	66	
Morrogh *et al*. 2012 [77]	11	100	100	0

*ER* estrogen receptor, *PR* progesterone receptor

Expression of PR is regulated by ER and is considered a prognostic marker of IBC [50]. Loss of PR expression is associated with endocrine resistance [51], and luminal B tumors are more often PR-negative/low compared to less aggressive luminal A tumors. Approximately 47 to 90 % of LCIS lesions express PR. However, the expression of PR is lower in LCIS lesions associated with IBC [43–46], and in apocrine PLCIS [31], implying that PR-low lesions are more likely to progress to invasive cancer [52]. Intriguingly, a recent study showed an inverse relationship between ER/PR status and Ki67 proliferation rate in ductal cancer but not in lobular cancer, such that ER-negative status did not correlate with high Ki67 in invasive lobular cancers whereas it did with invasive ductal cancers [52]. However, this has not yet been studied in detail in LCIS.

In contrast to ERα, the role of ERβ1 and the spliced variant ERβ2 in breast cancer is less well understood [53]. Some hypothesize that tamoxifen is an agonist of ERβ [54], thus suggesting that ERβ could be a marker of poor prognosis, due to its ability to oppose the anti-proliferative effects of tamoxifen-binding ERα. Recently, Huang and colleagues [55] measured ERβ expression in DCIS, invasive ductal cancer (IDC), and ILC. They concluded that while ERβ expression is high in normal mammary epithelial cells, ERβ expression is low in DCIS and IDC. In contrast, ILC tumors express higher levels of ERβ, with a reduction in expression in late stage ILC. LCIS samples were not included in this study. Results from an earlier study showed that LCIS has higher ERβ2 expression compared with normal epithelium, but that ERβ1 expression is not different [43]. Huang and colleagues concluded that the spliced variant ERβ2 is an indicator of hypoxia, not malignancy, which may explain the increased ERβ2 spliced variant in LCIS [43, 55]. In contrast to this observation, Nonni and colleagues [56] showed that ERβ expression in LN is significantly lower than in normal epithelium, although this study had a smaller sample size (n = 30).

Amplification of c-erbB-2 (HER2) is a marker of poor prognosis in patients with IBC. Fortunately, anti-HER2 antibodies have been effective drugs for HER2-positive tumors [57]. Understanding expression levels of HER2 in LCIS may shed light on its malignant nature. In LCIS, 0 to 11 % of tumors have HER2 amplification (Table 1). More aggressive LCIS subtypes are more likely to have amplified HER2 [44, 45]; 18 % of FLCIS and 31 % of apocrine PLCIS show HER2 amplification [33, 49].

Ki-67 expression is a marker of the proliferation rate of a tumor, and higher proliferative rates correlate with poor clinical outcomes [58]. In many LCIS lesions, Ki-67 expression is very low, corresponding to a 0 to 2 % proliferation rate in some studies [44, 45]. Other studies have shown that some LCIS lesions express a higher than 10 % proliferation rate [59]. Patients who have LCIS with higher proliferation rates may have a higher probability of relapse after surgery [59]. Currently, however, Ki67 is not used clinically to guide management decisions for LCIS.

The tumor suppressor gene encoding p53 is often dysregulated in human cancers [60]. In LCIS, p53 overexpression (reflecting protein stabilization as result of mutation) has been shown to be relatively low, ranging from 0 to 19 % using immunohistochemistry [44, 46]. Although the mutation rate of the p53 gene has not been assessed for LCIS, loss of heterozygosity has been observed for chromosome 17p, which is the location of the gene that encodes p53.

Recently, in a study by Andrade and colleagues, 23 patient-matched samples of normal breast tissue, LCIS, and ILC were subjected to microarray analysis to determine which genes might be involved in the progression of LCIS [61]. They identified 169 candidate genes involved in LCIS progression. The same study also showed that 40 CLCIS patient samples clustered in two groups, suggesting heterogeneity between CLCIS lesions at the transcriptomic level, even if they may otherwise appear homogenous.

The prognostic markers mentioned above do not reliably and accurately predict the potential of LCIS lesions to progress to invasive disease. Therefore, there is a critical need to identify better markers of progression, which might be used clinically to guide management.

### Genomic changes

Much of what is known about LCIS has been generated from studies utilizing aCGH. These studies, and others, suggest that LCIS and ILC are genetically similar and clonally related [20, 22, 23, 34, 62]. aCGH studies have also revealed similarities between lobular lesions and other low-grade lesions, including flat epithelial atypia, atypical ductal hyperplasia, low-grade DCIS and low grade IDC [34, 63, 64]. In light of these data, some have proposed that a broadly defined low-grade family of breast neoplasia exists, which has similar molecular drivers during disease progression [65, 66]. Characterization of breast cancer subtypes using gene expression profiling and DNA copy number variation has led to depiction of HER2-positive and ‘triple negative’ breast cancers as part of a ‘high-grade pathway’ and certain low-grade ER/PR-positive breast cancers as part of the ‘low-grade pathway’ [67]. Recently, this ‘low-grade precursor hypothesis’ has been challenged, with evidence that LCIS can progress into both low-grade and high-grade tumors [22, 67] and that LCIS can be a precursor to both ILC and IDC [62].

Specific chromosomal alterations are found frequently and consistently in LCIS. The chromosomal changes most commonly associated with LCIS are loss of 16q and gain of 1q [34]. Chromosome 16q contains several tumor suppressor genes, including E-cadherin (CDH1), a member of the calcium-dependent adhesion family of transmembrane proteins. Loss of other genes on 16q, including those encoding dipeptidase 1 (DPEP1) and CCCTC-binding factor (CTCF), have also been implicated in ILC [34, 68]. Loss of chromosome 16q, combined with mutations often resulting in premature stop codons and thus truncated proteins, transcriptional repression, and possibly gene promoter methylation, can lead to biallelic inactivation of CDH1. In addition to the 16q- and 1q + signature, many LCIS (both classical and pleomorphic) lesions demonstrate loss of 17p, which maps the gene encoding p53 [33]. Loss or amplification of 11q (containing the cyclin D1 gene) and loss of 8p are seen with a higher incidence in PLCIS compared with CLCIS. Furthermore, some FLCIS harbor amplification of 17q (spanning the gene encoding HER2), a finding seen less commonly in CLCIS [33]. Losses of 16p, and gains of 6q are also sometimes observed in LCIS [9]. Amplification of 16p and losses of 3q, 11q and 13q have also been described [49]. Results of aCGH experiments have shown that while most chromosomal changes in LCIS are not consistent, those that are most consistent (namely, 16q loss and 1q amplification) are found early in the progression to invasive disease. Although this information can be helpful for determining the relatedness of different lesions, it is less likely to be helpful clinically in distinguishing LCIS lesions that will progress from those that will not. Employing modern genomic techniques, such as next generation sequencing, will be critical in expanding our understanding of the genetic changes involved in the progression of LCIS.

LCIS is often multicentric, sometimes arising from 10 or more foci [69], and bilateral LCIS is also common. Furthermore, according to one study, about 23 % of women who develop LCIS have at least one first-degree relative with IBC [70]. Consistent genomic changes in LCIS may shed light on the genetic inheritance of the disease. There is evidence that germline polymorphisms in the *CDH1* gene (E-cadherin) predispose women to LCIS [71], and LCIS was also found in some patients with CDH1-related hereditary diffuse gastric cancer syndrome [72].

Recently, Sawyer and colleagues [73] analyzed SNPs in a total of 6539 lobular cancers, including 436 cases of pure LCIS, to identify those which specifically predisposed women to lobular disease. This study, which is part of GLACIER, a UK study of lobular breast cancer, utilized the iCOGS chip, a custom SNP array that comprises 211,155 SNPs enriched at predisposition loci for breast and other cancers [74]. Six SNPs were found that were strongly associated with ILC and LCIS, but not with IDC, with rs11977670 (7q34) showing the strongest association. Preliminary data in this study suggest that this SNP may influence levels and/or activity of JHDM1D, or SLC37A3, proteins with histone demethylase and sugar-phosphate exchanger functions, respectively. It is also possible that this SNP interacts with expression or function of the nearby *BRAF* gene, or that it controls expression of other non-coding genes. ENCODE data show overlap of the SNP with an area of H3K27 acetylation, supporting a role of this region in gene regulation. A SNP in LGR6 (rs6678914) showed specific associations with LCIS, and not with ILC. Similarly, other variants had stronger effect sizes in LCIS compared with ILC - for example, SNPs at *TOX3*, *ZNF365* and *MLLT10* loci. There were also SNPs that were more strongly associated with ILC compared with LCIS, including variants in the *FGFR2* and *MAP3K1* genes. Intriguingly, none of the 56 *CDH1* SNPs present on the iCOGS chip showed significant association with lobular cancer. This study provided an outstanding starting point for further functional studies of the identified pathways, especially to decipher their roles in development and progression of LCIS.

### Epithelial to mesenchymal transition markers in lobular carcinoma *in situ*

EMT is a process by which epithelial cells gain characteristics of mesenchymal cells, thereby promoting motility through tissue stroma [75, 76]. It has been proposed to be an essential step in breast cancer progression and metastasis. A critical component of EMT is the reduced function of cell-cell junctions, and it is feasible that EMT could also play a role in the development of LCIS, which is characterized by decreased cohesiveness within the lobule. Decreased expression of E-cadherin and dissociation of the cadherin-catenin complex is both a necessary step of EMT and a hallmark of lobular disease. E-cadherin loss and accumulation of cytosolic p120 catenin are frequently used diagnostically to differentiate between lobular and ductal lesions [11, 77]. A combination of mechanisms has been shown to contribute to the loss of E-cadherin, including somatic mutations, chromosomal loss, epigenetic silencing, and transcriptional repression (Table 2). The tight junction protein claudin 4, which plays a role in loss of cellular adhesion during EMT, was also shown to be downregulated in LCIS compared with normal tissue [78], and might thus also contribute to the decrease in cellular adhesion in lobular disease.

**Table 2:** Mechanisms of E-cadherin loss

Reference	Mechanism	Sample size	Percentage E-cadherin loss
Vos *et al*. 1997 [97]	LOH	7	86 %
Palacios *et al*. 2003 [98]	LOH	7	100 %
Sarrió *et al*. 2003 [99]	LOH	9	100 %
Mastracci *et al*. 2005 [100]	LOH	13	15 %
Vos *et al*. 1997 [97]	Mutation	2	100 %
Rieger-Christ *et al*. 2001 [101]	Mutation	21	38 %
Sarrió *et al*. 2003 [99]	Mutation	3	100 %
Mastracci *et al*. 2005 [100]	Mutation	14	100 %
Sarrió *et al*. 2003 [99]	Methylation	9	11 %
Morrogh *et al*. 2012 [77]	Transcription	36	100 %

*LOH* loss of heterozygosity

EMT has been shown to be driven by intrinsic transcription factors, such as SNAIL, SLUG, TWIST, and ZEB1, and by paracrine signaling molecules, including TGF-β and Wnt [76, 77, 79, 80]. In a subset of LCIS, some EMT genes, such as TWIST, are expressed [77, 81]. There is some evidence that in normal epithelial tissues, TWIST is epigenetically silenced through hypermethylation of its promoter region and its overexpression in LCIS is at least in part a result of hypomethylation [81]. TWIST expression is increased even more so in ILC [77], and high expression of ZEB1 was reported in poorly differentiated ILC [79, 80]. Thus, TWIST and ZEB1 may play a role in the development of ILC by promoting EMT through two major steps: dissociation of cell junctions with loss of polarity, and cytoskeletal changes that promote motility [76]. Another aspect of EMT involves cytoskeletal changes and increased motility [76]. Rho-GTPases control actin remodeling and are regulated by p120 catenin [82]. With the accumulation of cytosolic p120 catenin in lobular cancer, it is not surprising that p120 appears to be a major driver of the lobular phenotype [83]. LCIS cells demonstrate an affinity to interact with extracellular matrix components by increasing mesenchymal surface molecules like N-cadherin [84] and laminin receptor 1 [85]. Matrix metalloproteinase 9, well known to cause degradation of extracellular matrix to promote migration into stroma, was shown to be highly expressed in LCIS compared with normal mammary epithelium [78]. Collectively these data suggest the early LCIS lesions are poised for invasion; however, most will not progress to invasive disease.

### Activation of other signaling pathways in lobular carcinoma *in situ*

Several signaling pathways are commonly altered in lobular cancer. Perhaps most frequently, PIK3CA activating point mutations, long implicated in tumorigenesis, are found in both *in situ* and invasive lobular [86, 87]. In fact, in one study, 44 % (7 of 16 cases) of lobular neoplasias harbored activating PIK3CA mutations. Such mutations are also found in ductal cancers, and are not unique to breast carcinoma. As a comparison, these point mutations were found in 10 out of 21 (48 %) cases of DCIS and 13 out of 37 (35 %) invasive carcinomas [86].

Similarly to a variety of cancers, c-Src was found to be activated in both LCIS and ILC. Interestingly, some c-Src downstream targets such as Fak and Stat-3 were only active in ILC, but not in pre-invasive lobular neoplasia [84, 88]. Such activation thus represents a possible switch to allow LCIS cells to invade. In addition to Stat3, there is also some evidence for Stat5a playing a potential role in LCIS development and progression [89]. Stat5 is an important signaling molecule in the development of normal milk-producing mammary cells, and provides survival signals to mammary epithelial cells during lactation [90]. There is also evidence that increased Stat5 levels prevent apoptosis normally initiated by oncoproteins and involution [91]. Bratthauer and colleagues [89] reported strong staining for STAT5a in normal mammary epithelial cells, but loss in DCIS and IDC. Intriguingly, LCIS and ILC lesions retained STAT5 expression in 32 % and 17 % of the samples, respectively [89]. Amplification of prolactin receptor - an upstream activator of STAT5a signaling in breast tissue - is also observed in LCIS and ILC lesions, but not in DCIS lesions [92, 93]. These data suggest that STAT5a might provide survival signals to neoplastic cells in LCIS.

And finally, there is a report showing that cyclooxygenase-2 (COX-2) localizes within calveolae-like structures in the membrane, especially in more low-grade lesions [94, 95]. COX-2 expression has been implicated in the development of cancers by promoting an inflammatory environment conducive to tumor development [96] and, despite limitations, COX-2 inhibition may hold promise for cancer therapy and prevention. Further studies are necessary to understand the role of COX2, and in more general the role of the immune environment on development and progression of LCIS.

## Conclusion

LCIS is a clinically significant lesion which is incompletely understood and vastly understudied. Histological characteristics are the current standard for determining likelihood of progression of LCIS to IBC. It is likely that certain patients are either under- or overtreated. There is a critical need for better predictors of progression to invasive disease. The key to determining whether a LCIS lesion will progress will lie in the molecular characteristics of the lesion, including genetic aberrations in important signaling pathways, and alterations in EMT pathways. Since there are currently no models available to study LCIS, the generation of *in vitro* and *in vivo* model systems faithfully recapitulating the disease should be a focus of ongoing and future studies. A deeper understanding of the drivers of LCIS toward progression to invasive cancer may illuminate possible diagnostic targets that can allow clinicians to differentiate benign pre-invasive lesions from potentially malignant ones.

## Note

This article is part of a series on *Lobular breast cancer*, edited by Ulrich Lehmann. Other articles in this series can be found at http://breast-cancer-research.com/series/LBC.

## Abbreviations

aCGH: array comparative genome hybridization
ALH: atypical lobular hyperplasia
CLCIS: classical lobular carcinoma *in situ*
COX-2: cyclooxygenase-2
DCIS: ductal carcinoma *in situ*
EMT: epithelial to mesenchymal transition
ER: estrogen receptor
FLCIS: florid lobular carcinoma *in situ*
IBC: invasive breast cancer
IDC: invasive ductal cancer
ILC: invasive lobular carcinoma
LCIS: lobular carcinoma *in situ*
LIN: lobular intraepithelial neoplasia
LN: lobular neoplasia
PLCIS: pleomorphic lobular carcinoma *in situ*
PR: progesterone receptor
SNP: single nucleotide polymorphism
TDLU: terminal duct lobular unit
